# Expertise from the humanities and social sciences is essential for governmental responses to COVID-19

**DOI:** 10.7189/jogh.11.03081

**Published:** 2021-06-30

**Authors:** Martyn Pickersgill, Matthew Smith

**Affiliations:** 1 University of Edinburgh, Edinburgh, UK; 2 University of Strathclyde, Glasgow, UK

There can be few people untouched by the social and economic turbulence of the SARS-CoV-2 pandemic, even if they or their close ones have been medically unaffected by the virus. We have seen both the huge cost to human life of COVID-19 around the world, and the very considerable costs to lives as lived [1]. The disruption and abruption of swathes of educational, religious, and cultural activities and much, much more speak to the dramatic personal and political ramifications of managing a disease which at the start of last year did not even have a name [2].

The disastrous implications of the global spread of the SARS-CoV-2 virus have exacerbated and generated humanitarian crises in both classic and less familiar forms [3]. Increasing numbers of people, including in relatively affluent countries, continue to struggle to access the resources and support they need [4]. Despite this, expert advice and insight from the disciplines precisely concerned with the nature of societies and the workings of social, cultural, and economic processes – namely, the humanities and social sciences – have too often been under-utilised by policymakers tackling the pandemic.

SARS-CoV-2 is a virus and COVID-19 a serious disease, so of course expertise from biomedicine and public health is needed on national and international advisory groups aimed at addressing and managing the pandemic. However, the broader effects of COVID-19 also need more sustained attention [5]. SARS-CoV-2 permeates society and culture, undermining diverse aspects of well-being beyond a purely biomedical understanding of health. Innovative solutions to its management can consequently be derived by exploring historical and international experiences and re-examining and building from insights from philosophy and the arts [6].

Many of the questions that policymakers at local, national, and global levels are dealing with in relation to COVID-19 have significant social, economic, and ethical dimensions [7]. What, for instance, are both the immediate and longer-lasting implications of different exit strategies to stay-at-home mandates? How can individuals, communities, and infrastructures best recover from the pandemic and understand, anticipate, and adapt to the widespread changes to personal and professional lives that will result? And how can societies learn the lessons that will help us mitigate the effect of future pandemics and become fairer, more progressive, more equitable, and more sustainable? These questions demand close governmental collaboration and consultation with humanities and social scientific experts who have much to say and more to offer [8,9].

**Figure:**
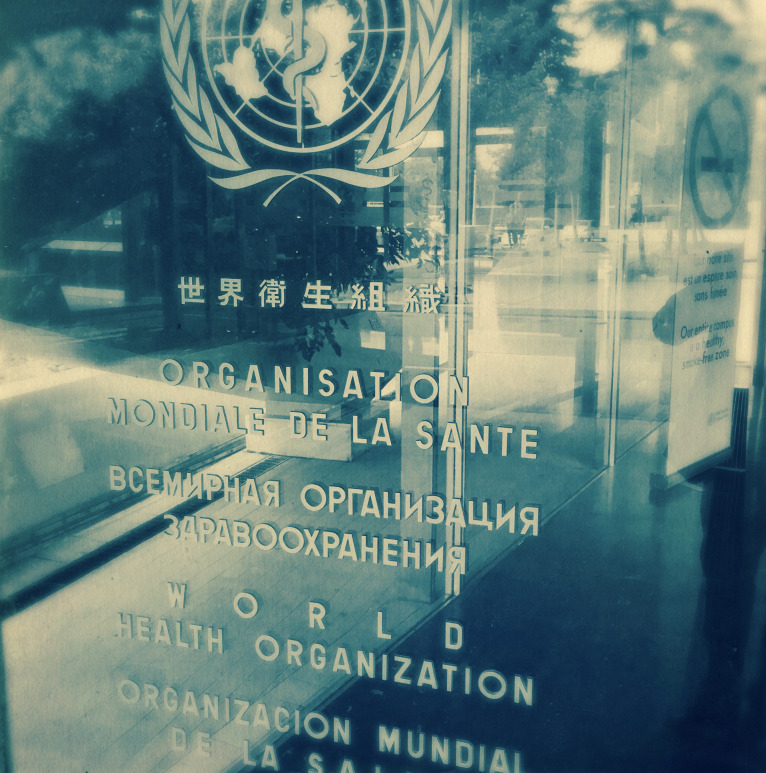
Photo: From Martyn Pickersgill’s collection, used with permission.

Like their colleagues within the biomedical and public health sciences, experts from the humanities and social sciences have, to-date, promoted the visibility and uptake of disciplinary insights to inform the management and recovery phases of societies, and have synthesised and put forward existing research evidence and insights to support decision-making. Experts from these disciplines have made their presence felt in multiple ways across the world. From carrying out vital research, to engaging with communities, to informing policy, there’s no vacuum of expertise.

At an international level, organisations like the World Health Organization (WHO) engages closely with such experts. Ethics scholars especially have informed various reports and briefings [10,11], with the WHO Working Group on Ethics and COVID-19 representing one key source of advice and expertise. At an early point in the pandemic the WHO also convened an international COVID-19 Social Science Working Group [12]. Similarly, a range of national governments have incorporated (to greater or lesser degrees) humanities and social science expertise within their broader pandemic advisory committees.

Yet, it remains the case that humanities and social science expertise can too easily be elided by politicians. This is perhaps not least when it complicates solutions deemed by others to be straightforward, or when those it offers are regarded as insufficiently resonant with the mores of powerful decision-makers. Further, while many experts in ethics, sociology, and other specialisms might feature as part of advisory committees, their numbers are generally low in comparison to their more visible colleagues in biomedicine and public health.

This has implications for the extent to which expertise from the humanities and social sciences can be successfully leveraged to augment – and, when necessary, to challenge – other inputs into policy. Public health decision-making also frequently rests on assumptions about social processes and cultural activities (about, for example, spirituality, mobility, and family composition, and practices). When moving at speed, the complexities of these are so vital to understand can be elided. If experts in the humanities and social sciences do not have a platform from which to, for instance, highlight the gaps between assumptions and realities, then limits to the success of COVID-19 management strategies will be inadvertently imposed.

Societies are formed through the interactions between histories, laws, traditions, and social relationships at different levels. Deep understandings of these and the complexity of their effects on economies, communities, and individuals are absolutely vital to informing the design, development, and roll-out of policy, regulatory, and technical interventions into societies afflicted by – and eventually recovering from – the SARS-CoV-2 virus. It is these understandings that must be brought to bear on the current crisis through an enhanced role for the humanities and social sciences as part of policy advising and making, as societies around the world seek to mitigate the worst aspects of COVID-19 - and move towards recovery.
